# Understanding unintentional childhood home injuries: pilot surveillance data from Karachi, Pakistan

**DOI:** 10.1186/1756-0500-5-37

**Published:** 2012-01-19

**Authors:** Nukhba Zia, Uzma R Khan, Junaid A Razzak, Prasanthi Puvanachandra, Adnan A Hyder

**Affiliations:** 1 Department of Emergency Medicine, Aga Khan University, Karachi, Pakistan; 2 Department of International Health & International Injury Research Unit, Bloomberg School of Public Health, Johns Hopkins University, Baltimore, MD, USA

**Keywords:** Child injuries, Unintentional injuries, Child health, Pakistan

## Abstract

**Background:**

Childhood injuries, an important public health issue, globally affects more than 95% of children living in low-and middle-income countries. The objective of this study is to describe the epidemiology of childhood unintentional injuries in Karachi, Pakistan with a specific focus on those occurring within the home environment.

**Methods:**

This was a secondary analysis of a childhood unintentional injury surveillance database setup in the emergency department of the Aga Khan Hospital, Karachi, Pakistan for 3 months. The data was collected by interviewing caretakers of children under 12 years of age presenting with an unintentional injury to the emergency departments of the four major tertiary care hospitals of Karachi, Pakistan.

**Results:**

The surveillance included 566 injured children of which 409 (72%) injuries had taken place at/around home. Of 409 children, 66% were males and mostly between 5 and 11 years of age. Injuries commonly occurred during play time (51%). Fall (59%), dog bites (11%) and burns (9%) were the commonest mechanisms of injury. The majority of the children (78%) were directly discharged from the emergency room with predicted short term disability (42%). There were 2 deaths in the emergency department both due to falls.

**Conclusion:**

Childhood injury surveillance system provides valuable in-depth information on child injuries. The majority of these unintentional childhood injuries occur at home; with falls, dog bites and burns being the most common types of unintentional childhood home injuries. Specific surveillance systems for child injuries can provide new and valuable information for countries like Pakistan.

## Background

Childhood injury is an important public health issue globally. Over 875,000 children of less than 18 years of age die annually in the world as a result of injuries, 80% of these occur in low- and middle-income countries (LMICs) [1]. According to recent estimates the death rate of unintentional injuries in LMICs is 65 per 100,000 population compared to 35 per 100,000 population in high-income countries (HICs). Similarly, the rate of disability adjusted life years (DALYs) lost due to unintentional injuries in LMIC is 2,398 per 100,000 population compared with 774 per 100,000 population in HICs [2]. Estimates have shown that most of childhood unintentional injuries take place in and around the home, where children are generally believed to be very well supervised [1]. Children, especially younger ones, spend a significant proportion of their time at home which exposes them to various injury hazards at home [3,4]. These hazards include stairs and windows without safety grills, access to poisonous substances and pesticides, open water reservoirs, access to stoves place on floor, knives and medicine.

Pakistan, a low-income country, with an estimated population of 170 million of which 43% are children between 0 and 14 years of age, [5] is located in the Eastern Mediterranean Region. The mortality rate of unintentional injuries in the low-and middle-income countries of the Eastern Mediterranean Region is 45.7/100,000 children [1]. Injury is considered to be the fifth leading cause of loss of healthy life and second leading cause of disability in Pakistan [6,7]. The National Injury Survey of Pakistan (NISP) conducted in 1997-99 showed that the estimated injury rate for children under 15 years in Pakistan is about 35.3 per 1,000 person years [8].

Studies done in various countries of the Eastern Mediterranean Region have shown that unintentional childhood injuries are most common at home [9-11]. Previous work done in Pakistan has also shown that home is the most common place where unintentional childhood injuries occur [3,12-15]. However, there is very little information available related to the types and mechanisms of childhood home injuries.

The overall goal of this paper is to contribute to the understanding of child injuries occurring in homes in the low income country of Pakistan. The specific objectives are: to describe the epidemiology of unintentional childhood home injuries presenting to a hospital emergency department (ED) in Karachi, Pakistan; to explore the predicted outcomes; and to reflect on the utility of a childhood injury surveillance system collecting data on home injuries.

## Methods

This study is secondary analysis of a pilot surveillance study (Additional file 1) done on unintentional childhood injuries presenting to the emergency departments (EDs) of both public and private hospitals in Karachi, Pakistan [3]. Karachi is the largest city of Pakistan with an estimated population of about 16 million [5]. It is the provincial capital of Sindh province and has both rural and urban areas divided into 18 administrative units or towns. The primary study in Karachi was done in the EDs of three public and one private hospital from February - April 2007. These EDs not only cater to the population of Karachi but also the patients coming from outside the city.

For the primary study, a surveillance form was administered to the caretakers of the injured children who were brought to the ED of the four hospitals in Karachi. The case definition was "*any child of less than 12 years of age coming to the ED of one of the four hospitals of Karachi due to an unintentional injury*". The exclusion criteria included children presenting with intentional injury including; assaults (stabbing, gun-shot wounds, gang violence, and child abuse), sexual assaults, self-inflicted injury or injuries related to drugs or alcohol; child older than 12 years of age, any child without parent or legal guardian and children presenting without injuries. The surveillance questions were based on previous work done internationally and in Pakistan [12,16-18]. The questionnaire included questions related to age, gender; type, mechanism and place/time of occurrence of injury, activity at the time of injury, outcome of emergency department treatment and self-reported questions on basic safety interventions practiced by caretakers like use of child car seat, use of seat-belts, helmet use, supervision of the child during bathing, accessibility to hot liquids and objects, hazardous materials and medications.

The age of the child was noted in three categories: less than one year old, between 1-4 years and 5-12 years. The categorization was done because of differences in the pattern of injuries in these age groups [12,13]. The mechanisms of injury taken into consideration were falls, road traffic injuries, fire/burns, smoke inhalation, foreign body injury, poisoning, choking on food, animal bites, machinery incidents, near-drowning and injury from a stationary object. The data collectors were trained specifically for this study, and the consent form was translated into Urdu. Ethical approval for the primary study and this analysis was taken from the ethics committees of each hospital before the commencement of study.

For this secondary analysis, we defined a home injury according to International Classification of External Causes of Injuries as "*A person's usual residence including adjacent grounds" *[16]. In addition to the area within and around home, for this study we also looked at the injuries that took place in the area around home and street outside the home. In Pakistan children commonly play outside home in streets where there is traffic. Simple frequencies were run for types of childhood home injury in Karachi. Cross tabulation was done for the type of childhood home injury with age, gender, activity at the time of injury, outcome of injury and disability. The disability was assessed by the treating emergency department physicians and noted by the data collectors in the questionnaire.

## Results

**Figure 1:**
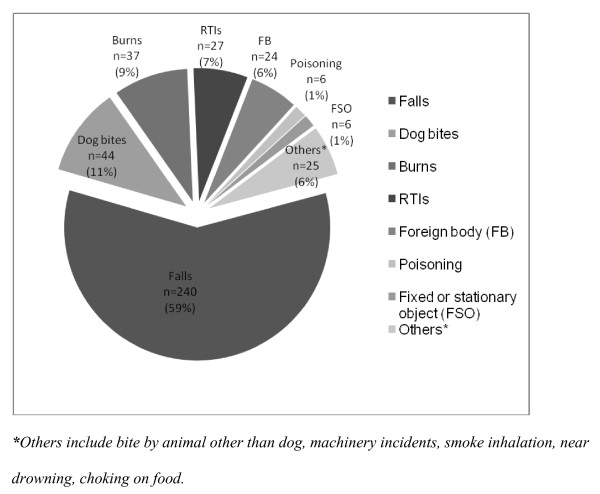
**Types of Unintentional Childhood Home Injuries (n = 409)**. *Others include bite by animal other than dog, machinery incidents, smoke inhalation, near drowning, choking on food.

Five hundred and sixty-six children less than 12 years of age were captured by the primary study with unintentional injuries in the EDs of the four hospitals in Karachi. Of the 566 cases, 409 (72%) injuries occurred at home, followed by those on the road (20%) and in school (5%) as shown in Table 1. The most common mechanisms of injury were falls (50%), road traffic injury (20%) and dog bites (11%). Figure 1 shows different types of unintentional childhood home injuries.

**Table 1:** Location of unintentional childhood injuries (n = 566), Karachi, Pakistan

Place of injury	n (%)
Home	409 (72)

Road/street	112 (20)

School	27 (5)

Sports/athletic area	7 (1.2)

Others*	11 (2)

Total	566

*Others places of injury included farm, trade/service area, public building

The most frequent causes of childhood unintentional home injuries were falls (n = 240, 59%) followed by dog bite (n = 44, 11%), burn injury (n = 37, 9%) and road traffic injury (n = 27, 6%). Of the 409 cases of home injuries, 221 (54%) children were between the ages of 5-11 years, and 168 (41%) between 1 and 4 years (Table 2). The overall male to female ratio was 1.9:1, with 66% of injuries in boys (Table 2). Unintentional home injuries mostly occurred during play time (n = 208, 51%).

**Table 2:** Childhood home injuries (n = 409) by age and gender in Karachi, Pakistan

	Injury mechanisms					
	
	Fall n (%)	Dog bite n (%)	Burns n (%)	RTIs n (%)	Others* n (%)	Total
**Age group**						

0-11 months	8 (3.3)	0	6 (16)	0	6 (10)	20

1 - 4 years	96 (40)	7 (16)	23 (62)	8 (30)	34 (56)	168

5 - 11 years	136(57)	37 (84)	8 (22)	19 (70)	21(34)	221

Total	240	44	37	27	61	409

**Gender**						

Male	158 (66)	33 (75)	22 (60)	21 (78)	36 (59)	270

Female	82 (34)	11 (25)	15 (40)	6 (22)	25 (41)	139

Total	240	44	37	27	61	409

*Others included injury due to foreign body, bite from an animal other than dog, machinery and stationary object, poisoning, drowning, smoke inhalation

Of the 240 cases of falls, 69 (29%) occurred from stairs/steps within home, 25 (10%) from bed and 18 (7.5%) from playground equipment within homes (for example play swings). There were 122 (51%) cases of fall from other heights for example wall, balcony, and fall on the same surface. There were 7 (3%) cases in which children sustained a fall from the hands of their attendants. In addition, there were two deaths due to falls; one was a fall from the first floor and the other was caused by fall into a sewerage tank (percentage mortality due to fall = 0.83%).

There were 37 cases of burns at home which affected more boys (n = 22, 59%) and children between the ages of 1-4 years (n = 23, 62%). The most common cause of burns was contact with hot liquids (n = 23, 62%); contact with hot objects (n = 5, 14%), fires (n = 2, 5%) and electrical burns (n = 2, 5%) represented a minority of cases.

Of the 27 cases of RTIs, the majority were male (n = 21, 78%) and children between 5 and 11 years of age (n = 19, 70%). RTIs occurred mostly when the children were either playing on the street outside their homes (n = 16, 59%). The most common striking vehicle was a motorcycle (n = 18, 67%), followed by mini-van/coaster (n = 4, 15%). There was one case in which the child was hit by a water tanker while he was playing outside his home.

Our study found 7 cases of poisoning; 4 occurred due to medicine ingestion, one each due to ingestion of a chemical, kerosene and spirit. Of these 7 cases, the poison agent was kept in water bottle (n = 2), beverage bottle (n = 2), jar (n = 1) and other non-labelled containers (n = 2). Two cases of near drowning were also reported. The vessel in which drowning took place included a bucket and a tub. Finally forty-four cases of dog bites were brought to the EDs, mostly boys (n = 33, 75%) and mostly children of 5-11 years of age (n = 37, 84%). Of the 44 cases, 19 (43%) were bitten by a dog while playing. All the cases of dog bites were discharged from the ED; 36 (82%) cases were expected to have short-term disability while one child was expected to have long-term disability.

Out of 409 cases, 319 (78%) were directly discharged from the ED, 49 (12%) required ward admission, 22 (5%) were transferred to other hospitals, 3 (0.7%) were detained in ED, 2 (0.5%) left against medical advice and one (0.2%) patient was referred to another hospital. The overall fatality rate due to all home injuries was 0.5% (n = 2) in the EDs. Disposition information related to 11 (2.7%) cases was missing.

Physicians in the ED predicted short-term disability ( < 6 weeks) as being likely in 170 (42%) cases and 72 (18%) were expected to suffer from long-term disability ( > 6 weeks). The surveillance systems had limited information on costs of treatment. About 37% (n = 150) of patients paid 'out of pocket' for their treatment and only 9% (n = 37) received welfare to cover the cost of their treatment; however there was no information related to the method of payment for the rest of 222 cases. The injuries in this study were mainly mild to moderate (n = 374, 91.5%); however 22 children had suffered serious to critical injuries.

When asked about the general safety precautions taken by the caretakers (self-reported) of children presenting with home injuries (n = 409), about 83.5% of the caretakers reported supervising their child while bathing. Around 23% had taken precautions to prevent access to hot liquids and 20% had taken steps to safely store hazardous material and medications away from the reach of the child. Only 4% of caretakers reported use of seat-belts while very few used age-appropriate car-seats (2%) or helmets (1%). Only 5% of caretakers placed their child on the back while sleeping to prevent suffocation.

## Discussion

Childhood injury surveillance can be conducted at multiple sites in a low income setting like Karachi, Pakistan and yield valuable insights into the nature and external causes of injuries. The home is the most common place where unintentional childhood injuries occur from surveillance data; as also observed in other ED-based studies (Additional file 1) [3,13,19,20]. Falls followed by dog bites and burns have been found to the most common mechanisms for these home injuries.

Community-based surveys on child injury prevention conducted by United Nations Children's Fund (UNICEF) and The Alliance for Safe Children (TASC) in six East and South Asian countries have shown that most of the non-fatal childhood injuries take place at home commonly among toddlers [4] One common hypothesis related to the high numbers of home injuries is that children spend significant amount of time at home especially in the younger ages; as a result they are exposed to a number of risks for home injury.

Our analysis showed that falls were the most common type of injury suffered by children at home accounting for more than half of the burden on EDs. This result is consistent with a recent study done in rural and suburban communities in Sindh and Balochistan provinces of Pakistan, which showed that about 51% of injuries in children between 1 and 8 years were attributed to falls [15]. Work done in other Eastern Mediterranean countries such as Syria and Iran on home injuries has also shown falls to be commonest injury suffered by preschool children [9,10,21]. A study done in Turkey on non-fatal unintentional home injuries stated that the percentage of children between 0 and 4 years, 5 and 9 years and 10 and 14 years suffering from falls to be 35%, 71.5% and 30% respectively [22]. Our study showed that the percentage of injury due to falls at home was increasing with the age, being highest in children between 5 and 11 years of age. The frequent causes of falls in our study were fall from steps/stairs followed by fall from bed.

In our pilot surveillance data dog bites is the second common home injury presenting to the EDs. This is consistent with some of the previous work and is very important locally [13,23,24]. Although there was no mortality due to dog-bite injury, many of these cases involved bites from stray dogs which are a common sight in Karachi due to lack of effective dog control measures. Dog bites have been reported as an important child health issue in Asia accounting for 56% of annual global deaths due to rabies, and represented a disease burden that is critical for both injury prevention and infectious disease (Rabies) control [25]. It is also a burden where the health sector of Karachi has to work effectively at local levels with civic administration and local government to control the problem of stray dogs efficiently. A National Dog Bite and Rabies Surveillance system is operational in Pakistan [26], funded by World Health Organization, to collect data on cases of dog bites in Pakistan.

As shown in previous studies, burns were in the leading three causes of unintentional home injury in children [10,15,21,22,27]. The youngest children were most affected (age group of 1-4 years) as also shown in previous studies [12-14,19] and the World report on child injury prevention [1]. The inquisitive nature of young children combined with their inability to comprehend harmful risk factors, and dependence on adult supervision makes them highly vulnerable to injuries.

This nexus of risks is also true for the cases of road traffic injuries that had taken place right outside the home of the victim. Majority of these injuries had taken place when the child was playing on the street and was hit by a motorcycle. The lack of play grounds, safe play areas, and lack of separation of traffic from the pedestrian zone is an ever present risk, especially in crowded urban areas like Karachi.

Like previous studies, [13,15,28] our results show that males represented a higher proportion of injured children compared to females. This has been attributed to higher risk-taking behavior in boys compared to girls; but in this age group and in our setting might represent other cultural issues as well. For example, studies in Pakistan have revealed a strong 'son preference' [29] and this has been reported to allow boys greater and earlier independence Also, there is likelihood that in our society injured male children are brought to the hospitals for treatment while females are mostly taken care of at homes.

Majority of the children in our study had suffered from mild to moderate injuries. Our study also revealed that there is lack of social protection and health insurance in Pakistan and patients have to pay out of their pockets for the treatment. This has grave implications on the family, as a vicious cycle of poverty may begin especially in cases of severe morbidity requiring long-term treatment and care.

This study used data from a pilot child injury surveillance system and like other facility based date has potential sampling bias as the study was conducted in only four hospital EDs in a city with a population of over 15 million people. Moreover, these hospitals are likely to see mostly severe cases of injuries; many minor to moderate injuries are treated either at home or by local community clinics. It is also sometimes difficult to assess the intent of injury in ED settings; for example, falls and burns in the database were categorized as unintentional injuries however; these may be intentional in nature. Thirdly, there is a likelihood that the number of fatalities seen in this study is an underestimation as many cases of death following an injury are not brought to the hospital due to medico-legal complications.

Caretakers reported a very small percentage using measures to prevent their children's accessibility to hot liquids, hazardous material and medications, or using age-appropriate car-seats and seat-belts. It is essential that caretakers are provided injury prevention information and access to such measures to prevent and control the exposure of children to the hazards of home injuries.

In addition, some important policy and implementation issues related to home injury prevention and control can be highlighted through this study. Firstly, it is important that in Pakistan standards for constructing houses should be followed. In the absence of such standards variability in the height and width of the steps/stairs constructed within homes is common. There is also generally lack of safety gates/grills/locks for the stairs; some houses may not even have a stair banister. In Pakistan, there is a common to build multi-level houses that further increase the risk of falls among children. Secondly, the local government needs to take measures to control the stray dogs present in the city. Thirdly, traffic calming and speed reducing measures needs to be enforced in the residential areas and around parks and playgrounds so that children do not risk their lives while playing on the roads and streets.

## Conclusion

The home environment is a common place for unintentional injuries among children presenting to hospitals in Karachi. Most home injuries are preventable through defining building standards/codes, controlling stray dogs, instituting traffic calming measures in residential areas, raise awareness through home visitation, pamphlet teaching and media campaigns. More focused research on the hazards/risks present in homes in Karachi need to be undertaken to develop specific prevention strategies.

## Competing interests

The authors declare that they have no competing interests.

## Authors' contributions

NZ performed analysis and interpretation of data and drafted the manuscript. URK carried out analysis and interpretation of data; helped in drafting the manuscript and revising it critically for important intellectual content. JR was involved in conception and design of the original study. He coordinated acquisition of data and interpretation and revised draft manuscript critically for important intellectual content and give final approval of the version to be published. PP was involved in conception and study design; revising draft manuscript critically for important intellectual content. AAH was involved in conception and study design; revising draft manuscript critically for important intellectual content and give final approval of the version to be published. All authors read and approved the final manuscript.

## Supplementary Material

Additional file 1 **Global childhood unintentional injury surveillance in four cities in developing countries: a pilot study**. Click here for file
