# Supplementary material for: Understanding unintentional childhood home injuries: pilot surveillance data from Karachi, Pakistan
Source: BMC Res Notes. 2012 Jan 19;5:37. doi: 10.1186/1756-0500-5-37 (PMC3337295; doi:10.1186/1756-0500-5-37)
Supplement: Additional file 1 — Global childhood unintentional injury surveillance in four cities in developing countries: a pilot study. [file 1756-0500-5-37-S1.PDF]

Global childhood unintentional injury surveillance in four cities in developing countries: a pilot study

Adnan A Hyder,[a] David E Sugerman,[b] Prasanthi Puvanachandra,[c] Junaid Razzak,[d] Hesham El-Sayed,[e] Andres Isaza,[f] Fazlur Rahman[g] & Margie Peden[h]

Objective To determine the frequency and nature of childhood injuries and to explore the risk factors for such injuries in low-income countries by using emergency department (ED) surveillance data.

Methods This pilot study represents the initial phase of a multi-country global childhood unintentional injury surveillance (GCUIS) project and was based on a sequential sample of children < 11 years of age of either gender who presented to selected EDs in Bangladesh, Colombia, Egypt and Pakistan over a 3–4 month period, which varied for each site, in 2007.

Findings Of 1559 injured children across all sites, 1010 (65%) were male; 941 (60%) were aged ≥ 5 years, 32 (2%) were < 1 year old. Injuries were especially frequent (34%) during the morning hours. They occurred in and around the home in 56% of the cases, outside while children played in 63% and during trips in 11%. Of all the injuries observed, 913 (56%) involved falls; 350 (22%), road traffic injuries; 210 (13%), burns; 66 (4%), poisoning; and 20 (1%), near drowning or drowning. Falls occurred most often from stairs or ladders; road traffic injuries most often involved pedestrians; the majority of burns were from hot liquids; poisonings typically involved medicines, and most drowning occurred in the home. The mean injury severity score was highest for near drowning or drowning (11), followed closely by road traffic injuries (10). There were 6 deaths, of which 2 resulted from drowning, 2 from falls and 2 from road traffic injuries.

Conclusion Hospitals in low-income countries bear a substantial burden of childhood injuries, and systematic surveillance is required to identify the epidemiological distribution of such injuries and understand their risk factors. Methodological standardization for surveillance across countries makes it possible to draw international comparisons and identify common issues.

Une traduction en français de ce résumé figure à la fin de l'article. Al final del artículo se facilita una traducción al español. الترجمة العربية لهذه الخلاصة في نهاية النص الكامل لهذه المقالة.

Introduction

Unintentional injuries are a leading cause of death among children and young adults.[1] Over 875 000 children ≤ 18 years of age die annually in the world as a result of injuries, mostly in low- and middle-income countries (LMIC), where injuries account for 13% of the total burden of morbidity among children ≤ 15 years of age.[2,3] According to a report from the United Nations Children's Fund, childhood injuries declined by 50% in high-income countries (HIC) between 1970 and 1995. Unfortunately, several reports from low-income countries have shown the opposite trend.[4–7]

The burden and pattern of child injuries are just now being studied in LMIC, where the age distribution of the population compounds the problem posed by child injuries. In 2005, 23% of the world's population of children < 5 years of age (i.e. 141 million children) lived in Africa, while only 10% lived in HIC.

In the global childhood unintentional injury surveillance (GCUIS) study, which was initiated in January 2007 in response to the lack of standardized child injury data from LMIC and the paucity of multi-country studies on child injuries, a standardized protocol was implemented to collect data from hospitals in several countries. The study objective was to determine the frequency and nature of childhood injuries in selected urban settings within LMIC by means of a pilot injury surveillance system. The pilot study described in this paper, which pools the results from four sites during the first phase of GCUIS, was intended to enhance the evidence base for planning child injury interventions in these areas. It is, to our knowledge, one of the first to collect standardized child injury data from emergency departments (EDs) in multiple sites in LMIC.

Methods

In the GCUIS study, a standard surveillance form was administered to the caretakers of injured children seen at EDs in four LMIC to identify all unintentional injuries. The surveillance questions were based on a variety of sources, including the *International classification of external causes of injuries* (in short form for pilot studies), the South African Red Cross

[a] Department of International Health, Johns Hopkins University Bloomberg School of Public Health, 615 North Wolfe Street, Baltimore, MD, 21205, United States of America (USA).
[b] Department of Emergency Medicine, Johns Hopkins Medical Institutes, Baltimore, MD, USA.
[c] International Injury Research Unit, Johns Hopkins Bloomberg School of Public Health, Baltimore, MD, USA.
[d] Department of Emergency Medicine, Aga Khan University, Karachi, Pakistan.
[e] Faculty of Medicine, Suez Canal University, Ismailia, Egypt.
[f] Universidad del Rosario, Bogotá, Colombia.
[g] Centre for Injury Prevention and Research, Dhaka, Bangladesh.
[h] Department of Violence and Injury Prevention and Disability, World Health Organization, Geneva, Switzerland.
Correspondence to Adnan A Hyder (e-mail: ahyder@jhsph.edu).
(*Submitted: 11 June 2008 – Revised version received: 6 November 2008 – Accepted: 1 February 2009*)

War Memorial Children's Hospital injury surveillance study instrument, and previous work done by the authors in Pakistan.[8–11] By using a standardized electronic data entry form in Epi Info version 3.3.2 (Centers for Disease Control and Prevention, Atlanta, GA, United States of America), we recorded the following for the GCUIS study: demographic information; data on mortality and disability from injuries and an injury severity score (ISS); risk factors such as age, gender, time of day and activity of the child when injured; selected cost of treatment; use of safety measures by families; and outcome of treatment in the ED. Details of the ISS used in this study, which provides an overall severity score for patients with multiple injuries, can be found elsewhere; in general, the lower the ISS score, the higher the probability of survival.[12,13] Physicians in the ED of the four participating sites were shown how to grade injury severity and were given comprehensive guidelines on using the surveillance instrument.[14]

In the GCUIS study, injury was operationally defined as any type of unintentional damage to any body part in patients seen in a designated GCUIS ED. The study focused on children < 12 years of age of either gender who presented to a designated ED in Bogotá, Colombia; Dhaka, Bangladesh; Ismailia, Egypt; or Karachi, Pakistan. Each participating hospital was a tertiary referral centre in an urban location and a general hospital for both children and adults. In each hospital data were collected for a 3–4 month period during 2007. To be selected for the study, each hospital had to be located in one of WHO's six world regions, engaged in ongoing collaborative research with the Johns Hopkins Bloomberg School of Public Health (JHBSPH) in Baltimore, MD, USA, and interested in participating. Excluded from the study were children who had suffered intentional injuries perpetrated by others (i.e. stabbings, gunshot wounds, other physical violence or sexual abuse), self-inflicted injury, or injuries related to drugs or alcohol; children ≥ 12 years of age; children without a parent or legal guardian; and children who presented to the hospital uninjured. The respondent for the surveillance system was the caretaker who accompanied the child to the ED, while the unit of analysis was the injured child.

Fig. 1. **Activity of children at time of injury (*n* = 1559): GCUIS data from four cities in Bangladesh, Colombia, Egypt and Pakistan, 2007**

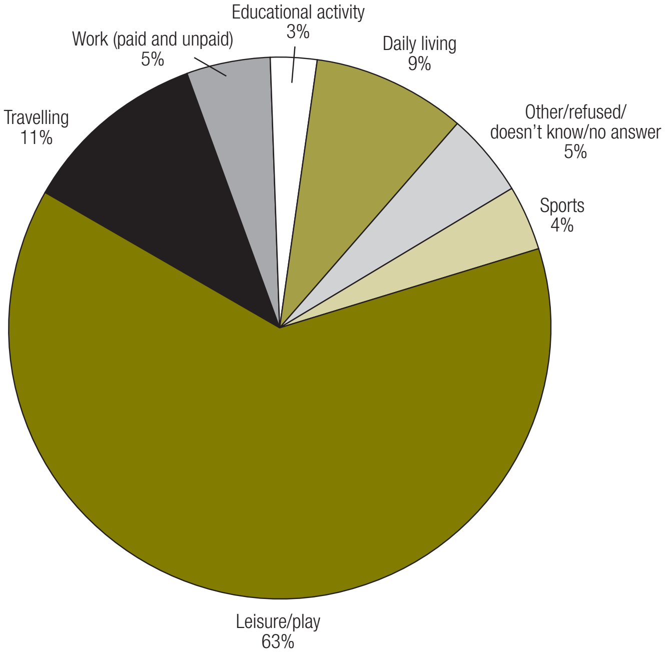

GCUIS, global childhood unintentional injury surveillance.

In all study sites, a pilot surveillance system with a common protocol was established, and the hospitals were requested to submit cases to the study coordinating centre at JHBSPH based on non-random, sequential sampling. A member of the team from JHBSPH visited each country and provided training on the surveillance system to the local collaborator. In addition, each site submitted the first 50 records on injured children to the coordinating centre for quality checks. Data from each country were recorded in Epi Info version 3.3.2 and then merged into a pooled database, which was subsequently cleaned, checked for errors and analysed with Intercooled STATA 7 (Stata Corp., College Station, TX, USA). Cross-tabulations of mode of injury by variables such as age, gender and time of injury were performed to provide greater insight into the determinants of unintentional injuries.

Data across four countries were pooled to describe the burden of injuries by means of simple frequencies. We examine the relationship between child injury and various factors surrounding the injury event and provide outcome data from each child's initial management in the ED. Ethical approval for this study was secured from the Institutional Review Board at the JHBSPH and from a research ethics committee in each hospital before the start of the study.

Results

The study sample consisted of 1559 children ≤ 12 years of age who were seen in hospital EDs in four urban centres in developing countries. Of these children, 32% were in Egypt, 28% in Pakistan, 25% in Bangladesh and 15% in Colombia. This paper focuses on an analysis of pooled data by type of injury. Of the total of 1559 cases, 1010 (65%) were male; 941 (60%) were ≥ 5 years of age, and 32 (2%) were < 1 year old. The observed injuries occurred throughout the day, but mostly between 06:00 and 12:00, and between 12:00 and 14:30 (34% and 21%, respectively); 63% of injuries occurred mainly while children were at play, while only 7% took place during school or other activities (Fig. 1).

The majority of the children arrived at the ED by taxi (33%) or private vehicle (28%), and a lesser proportion by ambulances (public or private) (Table 1). Most children were brought to the ED by their fathers (46%) or mothers (39%). Injuries that were not traffic-related occurred for the most part in and around homes. Children travelled an average of 20 km to the ED unless involved in a road traffic injury, in which case they travelled twice that

Table 1. **Descriptive data surrounding childhood injuries (*n* = 1559): GCUIS data from four cities in Bangladesh, Colombia, Egypt and Pakistan, 2007**

	RTI		Fall		Burn		Poisoning		Drowning		Total	
	No.	%	No.	%	No.	%	No.	%	No.	%	No.	%
**How was child transported to hospital?**												
Private car	86	25	263	29	44	21	27	41	11	55	431	28
Ambulance	107	31	110	12	33	16	9	8	1	6	260	17
Motorcycle	17	5	26	3	1	0	4	6	1	5	49	3
Bicycle	3	1	3	0	1	0	0	0	0	0	7	0
Walking	31	9	123	13	18	9	7	11	2	10	181	12
Taxi	80	23	303	33	109	52	10	15	5	25	507	33
Other	26	7	85	9	4	2	9	14	0	0	124	8
**Who took child to hospital?**												
Mother	111	32	376	41	78	37	35	53	6	30	606	39
Father	159	45	412	45	108	51	25	38	9	45	713	46
Other family member	51	15	92	10	22	10	6	9	5	25	176	11
Friend/teacher	7	2	21	2	2	1	0	0	0	0	30	2
Other	22	6	12	1	0	0	0	0	0	0	34	2
**Where did injury occur?**												
Home (inside and outside)	62	17	588	64	161	77	50	76	11	55	872	56
Road, street, highway	279	80	44	5	2	1	1	2	2	10	328	21
Countryside/farm/marketplace	2	1	9	1	1	0	0	0	1	5	12	1
Industrial building/other public building	2	1	157	17	44	21	13	20	6	30	222	14
School/sports/play area	3	1	96	11	2	1	2	3	0	0	103	7
Other/refused/unknown/no answer	2	1	19	2	0	0	0	0	0	0	21	1

GCUIS, global childhood unintentional injury surveillance; RTI, road traffic injury.

distance. The proportion of each type of injury was fairly constant across all four sites, with a preponderance of falls (56%), followed by road traffic injuries (22%) and burns (13%). For all types of injury, respondents (*n* = 1382) relied on government welfare or out-of-pocket payments for health-care expenses. Very few (1%) had private insurance for emergency care (Fig. 2).

The majority of injured children in this study were treated and discharged, but about one-third were admitted to the hospital (Table 2). A total of 71 children required emergency surgery, died in the ED or were transferred to another centre. Of the children discharged, 552 (36%) were expected to suffer short-term disability (< 6 weeks); 163 (11%), long-term disability (≥ 6 weeks); and 31 (2%), permanent disability. Overall ISSs were low (median, 4; mean, 7) across all sites and injury types.

Road traffic injuries

Of the 350 children involved in road traffic injuries, 39% were pedestrians, 19% were car passengers and 13% were motorcycle or moped riders (Table 3).

Fig. 2. **Primary method of payment for care received by injured children (*n* = 1382): GCUIS data from four cities in Bangladesh, Colombia, Egypt and Pakistan, 2007**

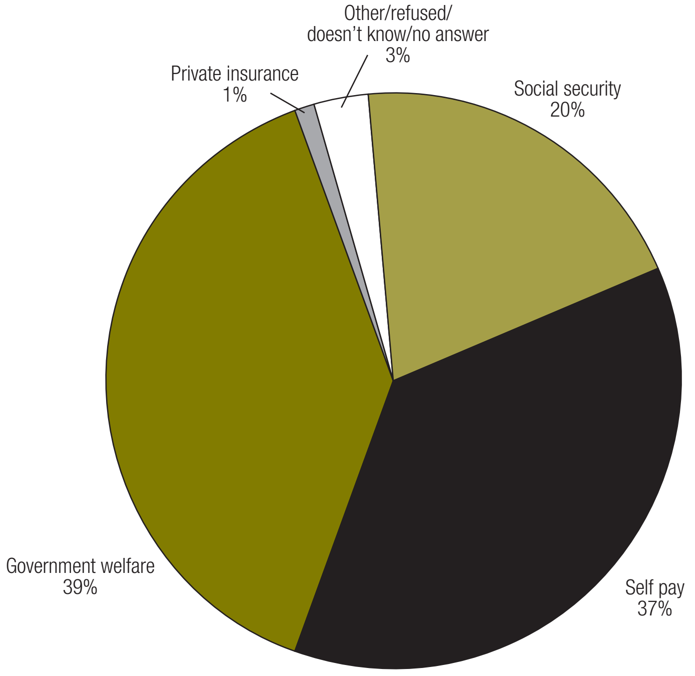

GCUIS, global childhood unintentional injury surveillance.

Table 2. **Characteristics of childhood injuries: GCUIS data from four cities in Bangladesh, Colombia, Egypt and Pakistan, 2007**

Characteristics	RTI		Fall		Burn		Poisoning		Drowning		Total	
	No.	%	No.	%	No.	%	No.	%	No.	%	No.	%
**Most severe injury**												
Fracture	65	21	195	25	0	0	1	2	1	6	262	19
Sprain	33	11	93	12	0	0	1	2	1	6	128	9
Cut/bite/open wound	78	25	200	25	0	0	1	2	7	39	286	21
Bruise/superficial	42	14	152	19	2	1	0	0	3	17	199	15
Burn	2	1	4	1	191	96	6	13	1	6	204	15
Concussion	80	26	134	17	1	1	0	0	3	17	218	16
Organ system injury	3	1	1	0	1	1	27	57	4	22	36	3
Other/refused/unknown/no answer	5	2	11	1	4	2	11	23	0	0	31	2
**Outcome of injury**												
Left before treatment completed	3	1	4	0	1	0	2	3	0	0	10	1
Referred to another centre	9	3	14	2	2	1	1	2	3	15	29	2
Treated and discharged home	173	55	717	79	41	20	52	79	11	55	994	66
Admitted to ward/burn unit/ICU	100	32	136	15	164	79	10	15	4	20	414	27
Admitted for emergency surgery	20	6	15	2	0	0	1	2	0	0	36	2
Died in ED	2	1	2	0	0	0	0	0	2	10	6	0
Other	7	2	20	2	0	0	0	0	0	0	27	2
**Projected effect of injury[a] (*n* = 1552)**												
No significant disability	119	38	483	53	108	51	53	80	13	65	776	51
Short-term temporary disability (< 6 weeks)	135	43	354	39	51	24	8	12	4	20	552	36
Long-term temporary disability (≥ 6 weeks)	52	17	70	8	35	17	5	8	1	5	163	11
Permanent disability	8	3	5	1	16	8	0	0	2	10	31	2
**Total**	**350**		**913**		**210**		**66**		**20**		**1 559**	

GCUIS, global childhood unintentional injury surveillance; ED, emergency department; ICU, intensive care unit; RTI, road traffic injury.
[a] As assessed by the treating physician.

Over 70% were males > 5 years of age, and the striking vehicles (315) were motorcycles (21%), cars (20%) and buses (19%). Of the children who suffered a road traffic injury, only 5% reported using seatbelts or helmets when injured. Road traffic injury cases had the highest percentage of projected long-term disability (17%) and 20 children required emergency surgery, representing the greatest need for emergency surgery across all causes of injury. The mean ISS for road traffic injury victims was 10. Two children died from their injuries (Table 2).

Falls

Falls were twice as common as road traffic injuries and the most common cause of injury (913, or 58.6%) among children who presented to the ED. They occurred mainly from ladders or stairs (23%), or from beds or other furniture (19%). Most falls occurred in males > 5 years of age and took place inside the home (38%) or during play (75%). The most severe fall-related injuries were fractures or open wounds. Children spent 3 hours on average in the ED (Table 2). Unlike children who suffered road traffic injuries, those who fell were generally treated and discharged with minimal disability or none. Two children died from fall-related injuries, for a proportionate mortality of 0.2%.

Burns

Of the 210 children with burns, 112 were injured by hot liquids, 41 by flames and 30 by electricity. Children of both genders were similarly affected, and 52% were < 5 years of age. Most burns occurred in the morning while the children played at home. Burned children remained in the ED an average of 1.5 hours. However, their admission rate (79%) was higher than that of children with other types of injuries. The mean ISS was 5.

Accidental poisoning

Children 1–4 years of age comprised the largest group of poisoning victims (56%), and males were most commonly involved (65%). Most of these children ingested medicines (32%), followed by cleaning agents (21%). The poisonings typically occurred in the morning while the children played at home. Organ system injury was the most severe and common consequence of poisoning (57%) (Table 2). Victims were typically sent home with limited disability or none (79%), and there were no deaths. Of the 1515 parents of poisoned children who replied to the question regarding storage, only 459 (30%) reported proper storage of hazardous materials at home.

Near drowning or drowning

Of the 20 children who drowned or nearly drowned, approximately half were male and 55% were > 5 years of age. Most children (85%) were playing outside their homes or in another building at the time of injury. Less than half of the parents reported supervising their children while bathing. Two children died in the ED, and the resulting proportionate mortality (10%) was higher than for any other injury type.

Discussion

This pilot study describes one of the first multi-country, ED-based injury surveillance systems for children in the developing world based on standardized methods. The results provide insight into the burden of childhood injuries on selected health facilities. In developing and implementing the GCUIS we learned that: (i) standardizing data collection is essential for comparing data on child injuries from different LMIC; (ii) new facility-based data collection systems can be developed and implemented in current EDs in different LMIC contexts; (iii) unless specifically included, childhood injuries are often ignored by routine hospital-based information systems; and (iv) country investigators are accomplished and capable of performing uniform protocol-based injury research in many LMIC.

The fact that a higher proportion of children who suffered road traffic injuries were pedestrians instead of vehicle passengers suggests that pedestrians of all ages are made vulnerable by sharing transport space with motorized vehicles. In studies from Pakistan, pedestrians and motorcyclists accounted for most of the injured and killed on the road, and 80% of all unintentional injuries and 67% of all resulting deaths in children were related to road traffic injuries.[15,16] Over half of road traffic injuries in Colombia occur in pedestrians,[17] while a study from Port-Said, Egypt, reported that pedestrians were involved in 81% of road traffic injuries.[18] The GCUIS study data also call attention to the frequent role of commercial vehicles in road traffic injuries in LMIC, where buses were the striking vehicle in a large proportion of childhood injury cases. In other studies, buses have been the most common striking vehicle, even though they represent a small proportion of all vehicles on the road.[15,16]

Although fall injuries were numerous in this study, they caused much lower morbidity than road traffic injuries, burns or poisoning. In many parts of the world, most childhood injuries treated in hospitals are due to falls that occur mainly at home.[19] Falls were the most common cause of injury in a study among school children in Ismailia, while in a study in Uganda, falls were the most common cause of severe injuries in children < 10 years of age.[20,21] In the Islamic Republic of Iran, falls were a leading cause of death among children < 1 year of age.[22] In Saudi Arabia, over 60% of orofacial injuries in children were due to falls[23], and an ED study in Trinidad and Tobago showed that falls accounted for 42% of all paediatric injuries.[24] The GCUIS study findings of a preponderance of early morning falls from stairs or ladders or from beds and other furniture while children were at play in and around the home suggests the need for a mixed intervention consisting of safer play areas, safer construction, safer furniture for sleeping and playing, and improved supervision.[25–27]

Table 3. **Road traffic injury data (*n* = 350): GCUIS data from four cities in Bangladesh, Colombia, Egypt and Pakistan, 2007**

Road traffic injury data	No.	%
**Mode of transit (*n* = 307)[a]**		
Passenger car	58	19
Three-wheeler	15	5
Bus (≥ 10 people)	15	5
Pickup/LDV/van/jeep/minibus (< 10 people)	8	3
Truck/heavy vehicle	0	0
Train	3	1
Motorcycle/moped	40	13
Animal or animal-drawn vehicle	3	1
Bicycle	29	9
Walk/run	121	39
Other	15	5
**Activity at impact (*n* = 148)[a]**		
Walking	18	12
Driving[b]	56	38
Passenger	53	36
Boarding vehicle	12	8
Riding on the outside of the vehicle	7	5
Other	2	1
**Striking vehicle or object (*n* = 315)[a]**		
Car	63	20
Three-wheeler	19	6
Bus/van	61	19
Truck/heavy car	18	6
Motorcycle	66	21
Bicycle	13	4
Animal	3	1
Fixed object	15	5
Other	57	18

GCUIS, global childhood unintentional injury surveillance; LDV, large domestic vehicle.
[a] Not all of the respondents whose children were involved in an RTI responded to these questions.
[b] Denotes children who were driving bicycles, mopeds or three-wheelers or prematurely driving cars and motorcycles.

Like other studies, the GCUIS study showed more frequent poisoning among males than females, predominantly with medicines and kerosene.[28] In the developing world, homes often store hydrocarbon, alkali, acid, and medicinal compounds used in cooking, cleaning, pest control and farming, and their improper storage puts children at risk of accidental ingestion.[29–31] Unintentional poisoning can be fatal without rapid treatment. Management requires intensive supportive care, provision of appropriate antidotes, if available, and removal of the substance from the body, all of which place substantial demands on the health-care system.[31,32]

In the GCUIS study, children who drowned or nearly drowned were almost equally divided among boys and girls. It may be that only a small number of the injured children presented to the ED because in LMIC most drowning victims die before reaching health facilities. The small number of parents who reported supervising their children while they bathed suggests a potential point of intervention.[33]

Children 1–4 years of age were disproportionately affected by burns and often required admission. Many studies have cited the risk to older children from cooking. However, in the GCUIS study, younger children were found to be at risk from playing near hot liquids at home.[34,35] A prospective case-control study from Bangladesh showed significant associations between burns and maternal illiteracy, pre-existing health impairments in children and low economic status.[36] An epidemiological review of burns highlighted the importance of certain risk factors (e.g. low maternal education and lack of supervision) within LMIC and the need to address them to enhance preventive efforts in these settings.[37] Such risk factors might differ among countries.

In the GCUIS study, the burden of severe injuries requiring hospitalization (27%) was higher than previously reported (2–10%).[38–41] Perhaps children who sustained only minor injuries did not seek treatment in the ED. Community-level data to explore this possibility was not, however, collected in the GCUIS study.

When parents have to leave work to take their injured children to the ED, or when they engage others to do it for them, the social cost is very high.[42,43] Injuries among older children who work in the informal sector to supplement the household income are economically disastrous. Over one-third of families (37%) paid for their care out-of-pocket because they lacked insurance. Such medical expenditures can be catastrophic and may tip households into poverty.[44] Another 39% of them relied on government or hospital welfare programmes, both of which increase the societal costs of managing child injuries.

A facility-based surveillance system like the GCUIS is advantageous when budgets are limited and time is a constraint, but its results cannot be extrapolated to the entire population, as the data are dependent on how often individuals seek care for injured children and thus subject to a host of financial, social and cultural factors that influence the decision to seek care.[45–47] Hospital-based data, especially in developing countries with lower rates of utilization, underestimate the injury burden. The results of this study were thus never meant to represent population-based morbidity from injuries. GCUIS focused only on unintentional injuries in children < 12 years of age, as a result of which the burden of intentional injuries (violence) and of injuries in children older than 12 years has not been captured. Age may have been misclassified in some cases, especially in children < 1 or 11–12 years of age.[48] Another shortcoming is the lack of ongoing ED surveillance at the study sites, which made it impossible to compare morbidity and mortality from childhood injuries and from other causes. Because GCUIS was implemented at different rates across sites, in each site the data were collected over different periods of 3–4 months in 2007, and seasonal variability may have influenced the results. This was a reality when working in LMIC. The GCUIS study did not include data from every region in the world and does not claim to represent all LMIC.

Capacity development was core to GCUIS implementation. Many sites had never participated in collaborative research, and some had never used statistical software. Technical assistance with data collection was ensured throughout this study. Site visits were important in the training of collaborators and database management and allowed for the development of good relationships and of a network of centres willing to continue working in the future.

Conclusion

The results of the GCUIS pilot study illustrate the feasibility of documenting the burden of childhood injuries on health systems in developing countries and of undertaking standardized child injury surveillance. They also suggest the need for tailored injury prevention research in LMIC, where the resulting data should encourage the conduct of interventional trials. The next step would be to implement appropriate injury prevention strategies, such as safe storage of medicines and cleaning supplies, protection of children from hot liquids, improved pedestrian safety, supervision during bathing, and use of stair-blocking gates. Ongoing child injury surveillance using standardized methods in LMIC is a tested strategy needed to track injuries and their risk factors and to monitor the impact of appropriate interventions.[49,50] ■

Acknowledgements

We thank Etienne Krug for his encouragement of the project, as well as all staff involved in data collection in all centres and all institutional officials who supported the conduct of the study.

Funding: This pilot study was funded by the Department of Violence and Injuries Prevention and Disability of the World Health Organization in Geneva, Switzerland.

Competing interests: None declared.

Résumé

Surveillance internationale des traumatismes non intentionnels de l'enfant dans quatre métropoles de pays en développement : étude pilote

Objectif Déterminer la fréquence et la nature des traumatismes touchant les enfants et étudier les facteurs de risque pour ces traumatismes dans des pays à faible revenu en utilisant les données de surveillance des services d'urgence.

Méthodes Cette étude pilote représente la phase initiale d'un projet multi-pays de surveillance des traumatismes non intentionnels de l'enfant (GCUIS) et a été menée à partir d'un échantillon séquentiel d'enfants de moins de 11 ans de l'un et l'autre sexe, qui ont été présentés dans des services d'urgence sélectionnés du Bangladesh, de Colombie, d'Egypte et du Pakistan, sur une période de 3-4 mois, variable selon les sites, au cours de l'année 2007.

Résultats Parmi les 1559 enfants victimes de traumatisme accueillis sur l'ensemble des sites, 1010 (65 %) étaient de sexe masculin, 941 (60 %) étaient âgés de 5 ans et plus et 32 (2 %) avaient moins d'un an. Les traumatismes étaient particulièrement fréquents (34 %) pendant la matinée. Ils se produisaient à l'intérieur et autour du domicile dans 56 % des cas, à l'extérieur pendant les jeux des enfants dans 63 % des cas et au cours de déplacements dans 11 % des cas. Sur l'ensemble des traumatismes observés, 913 (56 %) étaient liés à des chutes, 350 (22 %) à des accidents de la circulation, 210 (13 %) à des brûlures, 66 (4 %) à des empoisonnements et 20 (1 %) à des

« presque-noyades » ou à des noyades. Les chutes impliquaient le plus souvent des escaliers ou des échelles et les accidents de la circulation des piétons, la majorité des brûlures étaient dues à des liquides chauds, les empoisonnements faisaient intervenir habituellement des médicaments et la plupart des noyades se produisaient au domicile. Le score de gravité moyen des traumatismes était maximal pour les noyades et les presque-noyades (11), ce score étant suivi de près par celui des accidents de la circulation (10). L'étude a recensé 6 décès, dont 2 résultant d'une noyade, 2 d'une chute et 2 d'un accident de la circulation.

Conclusion Les hôpitaux des pays à faible revenu font face à une charge substantielle de traumatismes infantiles et une surveillance systématique est nécessaire pour déterminer la distribution épidémiologique de ces traumatismes et connaître les facteurs de risque associés. La standardisation méthodologique de la surveillance entre les pays permet de dresser des comparaisons internationales et d'identifier des problèmes communs.

Resumen

Vigilancia mundial de las lesiones infantiles no intencionales en cuatro ciudades de países en desarrollo: estudio piloto

Objetivo Determinar la frecuencia y naturaleza de las lesiones infantiles y explorar sus factores de riesgo en países de bajos ingresos, utilizando para ello los datos de vigilancia de los departamentos de urgencias (DU).

Métodos Este estudio piloto representa la fase inicial de un proyecto plurinacional de vigilancia mundial de las lesiones infantiles no intencionales y se basó en una muestra secuencial de menores de 11 años, de ambos sexos, que se presentaron en 2007 en DU seleccionados de Bangladesh, Colombia, Egipto y Pakistán a lo largo de un periodo de 3 a 4 meses, variable según el centro.

Resultados De 1559 niños lesionados observados en todos los centros, 1010 (65%) eran varones; 941 (60%) tenían ≥ 5 años, y 32 (2%) < 1 año. Las lesiones fueron especialmente frecuentes (34%) por la mañana. En el 56% de los casos se produjeron dentro de casa o en sus alrededores, en el 63% fuera, mientras jugaban, y en el 11% en viaje. De todas las lesiones observadas, 913 (56%) consistieron en caídas; 350 (22%) en lesiones causadas por el tráfico; 210 (13%) en quemaduras; 66 (4%) en intoxicaciones, y 20 (1%) en ahogamiento o casi ahogamiento. Las caídas más frecuentes se produjeron en escaleras; en el caso de las lesiones causadas por el tráfico, la mayoría de las víctimas fueron peatones; la mayoría de las quemaduras se produjeron con líquidos calientes; las intoxicaciones más frecuentes se debieron a medicamentos, y la mayoría de los ahogamientos se produjeron en casa. Las lesiones con mayores puntuaciones de gravedad fueron los ahogamientos o casi ahogamientos (11), seguidos de cerca por las lesiones causadas por el tráfico (10). Hubo 6 casos mortales: 2 por ahogamiento, 2 por caídas y 2 por lesiones causadas por el tráfico.

Conclusión Los hospitales de los países de bajos ingresos tienen una carga considerable de lesiones infantiles. Es necesaria una vigilancia sistemática para identificar la distribución epidemiológica de esas lesiones y comprender sus factores de riesgo. La normalización metodológica de la vigilancia en los diferentes países permite establecer comparaciones internacionales e identificar problemas comunes.

ملخص

لترصُّد العالمي للإصابات غير المتعمدة لدى الأطفال في أربع مدن من بلدانٍ نامية: دراسة ارتيادية

الهدف: تعيين تكرار وطبيعة الإصابات لدى الأطفال، واستكشاف عوامل الخطر الناجمة عن هذه الإصابات في البلدان المنخفضة الدخل باستخدام معطيات الترصُّد في أقسام الطوارئ.

الطريقة: تمثِّل هذه الدراسة الارتيادية المرحلة الأولى من مشروع الترصُّد العالمي المتعدد البلدان للإصابات غير المتعمدة لدى الأطفال، وقد ارتكز على عينة تتابعية من الأطفال دون سن الحادية عشرة من كلا الجنسين ممن أحضروا إلى أقسام الطوارئ في بنغلاديش وكولمبيا ومصر وباكستان على مدى 3-4 شهور تختلف باختلاف كل موقع وذلك في عام 2007.

الموجودات: بلغ عدد الأطفال في جميع المواقع 1559، منهم 1010 (65%) من الذكور، و941 (60%) دون خمس سنوات من العمر، 32 (2%) دون السنة الواحدة من العمر. وقد كانت الإصابات متكررة بشكل خاص (34%) أثناء ساعات الصباح، وهي تحدث داخل المنزل وحوله في 56% من الحالات، وخارج المنزل أثناء لعب الأطفال في 63% منهم وأثناء الرحلات لدى 11% منهم. ومن بين مجمل الإصابات الملاحظة، شمل 913 منها (56%) السقوط؛ 350 (22%) إصابات المرور على الطرق؛ و210 (13%) حروق؛ و66 (4%) تسممات؛ و20 (1%) الإشراف على الغرق أو الغرق. وكان الوقوع في معظم الحالات من الأدراج أو السلالم؛ وكانت الإصابات على الطرق غالباً ما تصيب مشاة؛ وكان معظم الحروق بالمياه الساخنة، والتسممات في معظم الحالات نتجت عن الأدوية، كما أن معظم حالات الغرق حدثت في المنزل. وكان وسطي درجة شدة الإصابة أعلى ما يكون في الإشراف على الغرق أو الغرق (11)، يتلوه الإصابات في الحوادث على الطرق (10) وكان هناك 6 وفيات، منها اثنتان نجمتا عن الغرق، واثنتان عن السقوط واثنتان عن الإصابات على الطرق.

الاستنتاج: تتحمل المستشفيات في البلدان المنخفضة الدخل عبئاً جسيماً جسيم من إصابات الأطفال، وتمس الحاجة إلى ترصد منهجي للتعرف على التوزيع الوبائي لمثل هذه الإصابات، ولفهم عوامل الاختطار. إن فرص المقاييس المنهجية للترصد على جميع البلدان يجعل بالإمكان إجراء مقارنات دولية والتعرف على القضايا المشتركة.

References

1. Krug EG, Sharma GK, Lozano R. The global burden of injuries. *Am J Public Health* 2000;90:523-6. PMID:10754963 doi:10.2105/AJPH.90.4.523
2. Murray CJ, Lopez AD. Alternative projections of mortality and disability by cause, 1990-2020: global burden of disease study. *Lancet* 1997; 349:1498-504. PMID:9167458 doi:10.1016/S0140-6736(96)07492-2

3. Deen JL, Vos T, Huttly SR, Tulloch J. Injuries and noncommunicable diseases: emerging health problems of children in developing countries. *Bull World Health Organ* 1999;77:518-24. PMID:10427938
4. *A league table of child deaths by injuries in rich nations* (Innocenti Report Card Series, issue no. 2). New York, NY: UNICEF; 2001.
5. Guastello SJ. Injury analysis and prevention in the developing countries. *Accid Anal Prev* 1999;31:295-6. PMID:10384220
6. Hyder AA, Wali S, Fishman S, Schenk E. The burden of unintentional injuries among the under-five population in South Asia. *Acta Paediatr* 2008; 97:267-75. PMID:18298772 doi:10.1111/j.1651-2227.2008.00670.x
7. *The world health report 2004: changing history*. Geneva: World Health Organization; 2004.
8. International Classification of External Causes of Injuries Coordination and Maintenance Group. *International classification of external causes of injuries, version* 1.2. Consumer Safety Institute and AIHW National Injury Surveillance Unit: Amsterdam and Adelaide; 2004.
9. Fatmi Z, Hadden WC, Razzak JA, Qureshi HI, Hyder AA, Pappas G. Incidence, patterns and severity of reported unintentional injuries in Pakistan for persons five years and older: results of the National Health Survey of Pakistan 1990-94. *BMC Public Health* 2007;7:152. PMID:17623066
10. Ghaffar A, Hyder AA, Masud TI. The burden of road traffic injuries in developing countries: the 1st national injury survey of Pakistan. *Public Health* 2004;118:211-7. PMID:15003410 doi:10.1016/j.puhe.2003.05.003
11. Lalloo R, van As AB. Profile of children with head injuries treated at the trauma unit of Red Cross War Memorial Children's Hospital, 1991-2001. *S Afr Med J* 2004;94:544-6. PMID:15285456
12. Baker SP, O'Neill B, Haddon W Jr, Long WB. The injury severity score: a method for describing patients with multiple injuries and evaluating emergency care. *J Trauma* 1974;14:187-96.
13. Copes W, Sacco W, Champion H, Bain L, eds. Progress in characterising anatomic injury. In: *Proceedings of the 33rd Annual Meeting of the Association for the Advancement of Automotive Medicine*; 1989:205-18.
14. Gennarelli TA, Wodzin E. AIS 2005: a contemporary injury scale. *Injury* 2006;37:1083-91. PMID:17092503 doi:10.1016/j.injury.2006.07.009
15. Luby S, Hassan I, Jahangir N, Rizvi N, Farooqi M, Ubaid S, et al. Road traffic injuries in Karachi: the disproportionate role of buses and trucks. *Southeast Asian J Trop Med Public Health* 1997;28:395-8. PMID:9444028
16. Razzak JA, Luby SP, Laflamme L, Chotani H. Injuries among children in Karachi, Pakistan — what, where and how. *Public Health* 2004;118:114-20. PMID:15037041 doi:10.1016/S0033-3506(03)00147-1
17. Posada J, Ben-Michael E, Herman A, Kahan E, Richter E. Death and injury from motor vehicle crashes in Colombia. *Rev Panam Salud Publica* 2000;7:88-91. PMID:10748658 doi:10.1590/S1020-49892000000200003
18. Hassan F, El-Sheikh E. Hospital-based surveillance of trauma in Port-Said. *Alexandria Med J* 1998;40:629-40.
19. Bangdiwala SI, Anzola-Perez E, Romer CC, Schmidt B, Valdez-Lazo F, Toro J, et al. The incidence of injuries in young people: I. Methodology and results of a collaborative study in Brazil, Chile, Cuba and Venezuela. *Int J Epidemiol* 1990;19:115-24. PMID:2351505 doi:10.1093/ije/19.1.115
20. El-Sayed H, Hassan F, Gad S, Abdel-Rahman A. Pattern and burden of injuries among school children in Ismailia city, Egypt. *Egyptian J Pediatr* 2003;20:201-10.
21. Kobusingye O, Guwatudde D, Lett R. Injury patterns in rural and urban Uganda. *Inj Prev* 2001;7:46-50. PMID:11289535 doi:10.1136/ip.7.1.46
22. Soori H, Naghavi M. Childhood deaths from unintentional injuries in rural areas of Iran. *Inj Prev* 1998;4:222-4. PMID:9788095 doi:10.1136/ip.4.3.222
23. Lawoyin TO, Lawoyin DO, Lawoyin JO. Factors associated with oro-facial injuries among children in Al-Baha, Saudi Arabia. *Afr J Med Med Sci* 2002;31:37-40. PMID:12518927
24. Kirsch TD, Beaudreau RW, Holder YA, Smith GS. Pediatric injuries presenting to an emergency department in a developing country. *Pediatr Emerg Care* 1996;12:411-5. PMID:8989787 doi:10.1097/00006565-199612000-00006
25. Butchart A, Kruger J, Lekoba R. Perceptions of injury causes and solutions in a Johannesburg township: implications for prevention. *Soc Sci Med* 2000;50:331-44. PMID:10626759 doi:10.1016/S0277-9536(99)00272-5
26. Tandon JN, Kalra A, Kalra K, Sahu SC, Nigam CB, Qureshi GU. Profile of accidents in children. *Indian Pediatr* 1993;30:765-9. PMID:8132256
27. Reichenheim ME, Harpham T. Child accidents and associated risk factors in a Brazilian squatter settlement. *Health Policy Plan* 1989;4:162-7. doi:10.1093/heapol/4.2.162
28. Fernando R, Fernando DN. Childhood poisoning in Sri Lanka. *Indian J Pediatr* 1997;64:457-60. PMID:10771874 doi:10.1007/BF02737748
29. Lang T, Thuo N, Akech S. Accidental paraffin poisoning in Kenyan children. *Trop Med Int Health* 2008;13:845-7. PMID:18363584
30. Konradsen F, Pieris R, Weerasinghe M, van der Hoek W, Eddleston M, Dawson AH. Community uptake of safe storage boxes to reduce self-poisoning from pesticides in rural Sri Lanka. *BMC Public Health* 2007;7:13. PMID:17257415 doi:10.1186/1471-2458-7-13
31. Meyer S, Eddleston M, Bailey B, Desel H, Gottschling S, Gortner L. Unintentional household poisoning in children. *Klin Padiatr* 2007;219:254-70. PMID:17763291 doi:10.1055/s-2007-972567
32. Eddleston M, Senarathna L, Mohamed F, Buckley N, Juszczak E, Sheriff MH, et al. Deaths due to absence of an affordable antitoxin for plant poisoning. *Lancet* 2003;362:1041-4. PMID:14522536
33. Brenner RA. Childhood drowning is a global concern. *BMJ* 2002; 324:1049-50. PMID:11991894 doi:10.1136/bmj.324.7345.1049
34. Poudel-Tandukar K, Nakahara S, Ichikawa M, Poudel KC, Joshi AB, Wakai S. Unintentional injuries among school adolescents in Kathmandu, Nepal: a descriptive study. *Public Health* 2006;120:641-9. PMID:16759678
35. Cuenca-Pardo J, de Jesus Alvarez-Diaz C, Compres-Pichardo TA. Related factors in burn children. Epidemiological study of the burn unit at the "Magdalena de las Salinas" Traumatology Hospital. *J Burn Care Res* 2008;29:468-74. PMID:18388578
36. Daisy S, Mostaque AK, Bari TS, Khan AR, Karim S, Quamruzzaman Q. Socioeconomic and cultural influence in the causation of burns in the urban children of Bangladesh. *J Burn Care Rehabil* 2001;22:269-73. PMID:11482685 doi:10.1097/00004630-200107000-00004
37. Forjuoh SN. Burns in low- and middle-income countries: a review of available literature on descriptive epidemiology, risk factors, treatment, and prevention. *Burns* 2006;32:529-37. PMID:16777340 doi:10.1016/j.burns.2006.04.002
38. Yamamoto LG, Wiebe RA, Matthews WJ Jr. A one-year prospective ED cohort of pediatric trauma. *Pediatr Emerg Care* 1991;7:267-74. PMID:1754484
39. Bienefeld M, Pickett W, Carr PA. A descriptive study of childhood injuries in Kingston, Ontario, using data from a computerized injury surveillance system. *Chronic Dis Can* 1996;17:21-7. PMID:9079349
40. Bener A, Al-Salman KM, Pugh RN. Injury mortality and morbidity among children in the United Arab Emirates. *Eur J Epidemiol* 1998;14:175-8. PMID:9556177 doi:10.1023/A:1007444109260
41. Sturms LM, van der Sluis CK, Groothoff JW. Duis HJt, Eisma WH. Characteristics of injured children attending the emergency department: patients potentially in need of rehabilitation. *Clin Rehabil* 2002;16:46-54. PMID:11837525 doi:10.1191/0269215502cr466oa
42. Spiegel DA, Gosselin RA, Coughlin RR, Joshipura M, Browner BD, Dormans JP. The burden of musculoskeletal injury in low and middle-income countries: challenges and opportunities. *J Bone Joint Surg Am* 2008;90:915-23. PMID:18381331 doi:10.2106/JBJS.G.00637
43. Gurses D, Sarioglu-Buke A, Baskan M, Kilic I. Cost factors in pediatric trauma. *Can J Surg* 2003;46:441-5. PMID:14680351
44. Aeron-Thomas A, Jacobs G, Sexton B, Gururaj G, Rahman F. *The involvement and impact of road crashes on the poor: Bangladesh and India case studies.* Crowthorne, UK: Transport Research Laboratory; 2004 (PR/INT/275/2004).
45. D'Souza RM. Care-seeking behavior. *Clin Infect Dis* 1999;28:234. PMID:10064233 doi:10.1086/515120
46. Thind A, Andersen R. Respiratory illness in the Dominican Republic: what are the predictors for health services utilization of young children? *Soc Sci Med* 2003;56:1173-82. PMID:12600356 doi:10.1016/S0277-9536(02)00116-8
47. Waterston T. Children's rights and child health: what role for paediatricians? *J Trop Pediatr* 2003;49:260-2. PMID:14604156
48. Bairagi R, Aziz KMA, Chowdhury MK, Edmonston B. Age misstatement for young children in rural Bangladesh. *Demography* 1982;19:447-58. PMID:7173466 doi:10.2307/2061012
49. *Child and adolescent injury prevention: a global call to action.* Geneva: World Health Organization, UNICEF; 2005. Available from: http://whqlibdoc.who.int/publications/2005/9241593415_eng.pdf
50. *Guidelines for conducting community surveys on injuries and violence.* Geneva: World Health Organization; 2004.
